# Motivations, barriers, and professional engagement: a multisite qualitative study of internal medicine faculty’s experiences learning and teaching point-of-care ultrasound

**DOI:** 10.1186/s12909-022-03225-w

**Published:** 2022-03-12

**Authors:** Christopher J. Smith, Keith Barron, Ronald J. Shope, Elizabeth Beam, Kevin Piro

**Affiliations:** 1 Department of Internal Medicine, Division of Hospital Medicine, University of Nebraska Medical Center, 986430 Nebraska Medical Center, Omaha, NE USA; 2 Prisma Health Midlands - University of South Carolina School of Medicine, Department of Internal Medicine, Division of General Internal Medicine, 14 Richland Medical Park Drive, Suite, Columbia, SC 320 USA; 3 Department of Health Promotion, University of Nebraska Medical Center, 984365 Nebraska Medical Center, Omaha, NE USA; 4 Interprofessional Academy of Educators, University of Nebraska Medical Center, 987115 Nebraska Medical Center, Omaha, NE USA; 5 Department of Internal Medicine, Division of Hospital Medicine, Oregon Health & Science University, 3270 SW Pavilion Loop Suite, Portland, OR 350 USA

**Keywords:** Point-of-care ultrasound (POCUS), Faculty development, Curriculum design, Qualitative research

## Abstract

**Background:**

Point-of-care ultrasound (POCUS) graduate medical education is expanding across many specialties, but a lack of trained faculty is a common barrier. Even well-designed faculty development programs struggle with retention, yet little is known about the experiences of practicing physicians learning POCUS. Our objective is to explore the experiences of clinician-educators as they integrate POCUS into their clinical and teaching practices to help inform curriculum design.

**Methods:**

Qualitative study using instrumental case study design to analyze interview data from 18 internal medicine clinician-educators at 3 academic health centers. Interviewees were recruited by program directors at each site to include participants with a range of POCUS use patterns. Interviews took place from July–August 2019.

**Results:**

Analysis yielded 6 themes: teaching performance, patient care, curriculum needs, workflow and access, administrative support, and professional engagement. Participants felt POCUS enhanced their teaching skills, clinical decision making, and engagement with patients. The themes highlighted the importance of longitudinal supervision and feedback, streamlined integration of POCUS into clinical workflow, and administrative support of time and resources. Interviewees reported learning and teaching POCUS helped combat burn-out and enhance their sense of professional engagement.

**Conclusions:**

Learning POCUS as a practicing clinician-educator is a complicated endeavor that must take into account mastery of psychomotor skills, existing practice habits, and local institutional concerns. Based upon the themes generated from this study, we make recommendations to help guide POCUS faculty development curriculum design. Although this study focused on internists, the findings are likely generalizable to other specialties with growing interest in POCUS education.

**Supplementary Information:**

The online version contains supplementary material available at 10.1186/s12909-022-03225-w.

## Background

Point-of-care ultrasound (POCUS) is the use of sonographic imaging by clinicians to assist in real-time patient care decisions. POCUS can augment the physical exam [1–3], improve diagnostic accuracy [4–7], and enhance procedural safety [8–10], leading to its broad adoption into medical school curricula [11, 12]. The influx of residents with POCUS training combined with more affordable and portable devices has contributed to an expansion of POCUS graduate medical education across various specialties [13–15]. This growth has been especially notable within internal medicine (IM). Between 2012 and 2016 the number of IM residency programs with formal POCUS training increased from 25% [16] to 40% [13], a trend that has likely accelerated in the intervening years. In 2019 the Alliance for Academic IM released a position statement supporting the integration of POCUS training across undergraduate, graduate, and continuing medical education [17].

While there is great enthusiasm for POCUS education, a dearth of trained faculty has been a persistent barrier to program expansion [13, 16, 18, 19]. Consequently, faculty development is a major area of need for nascent programs with the dual aims of supporting motivated colleagues and building mentoring networks for the growing number of POCUS learners. As with other practice changes, the process of learning POCUS is a complicated endeavor that is impacted by individual, social, and organizational factors [20]. Practicing physicians must master a complex set of psychomotor skills, integrate these skills within existing practice habits, adhere to organizational policies and procedures, and manage other competing responsibilities. Because of these issues, even well-designed POCUS faculty development programs can struggle with retention over time [21]. Thus, as the need for POCUS educators intensifies, it is becoming increasingly essential to have a detailed understanding of the factors impacting faculty learners.

The purpose of this study was to explore IM physicians’ experiences learning and teaching POCUS at 3 university-based health systems to help guide future curriculum design.

## Methods

### Design

We performed a multi-site qualitative study using instrumental case study design. Case study design is rooted in the constructivist paradigm and is best used to describe the “how” and “why” complex phenomenon occur within their real-life context. An instrumental case study focuses on understanding a specific issue rather than understanding individual cases [22]. We defined the case as the experiences of IM clinician-educators learning and integrating POCUS into their practice. Since this is a complex issue, we wanted to examine the issue across multiple sites using a holistic analysis [23] rather than comparing and contrasting individual sites. We bound the case definition by practice location (3 university-affiliated healthcare centers with IM residency programs), profession (IM physicians), and prior participation in POCUS faculty development at their home institution.

### Setting & participants

Table 1 lists characteristics of the 3 study sites. These sites were chosen because they had established faculty and resident POCUS training programs and were located in different geographic regions of the country. We used purposeful sampling to recruit physicians with a range of POCUS use patterns to participate in semi-structured interviews. Eligible participants were identified by POCUS program directors at each site (CS, KB, KP) based upon participants’ frequency of POCUS use (frequent and infrequent users) and recruited via email. Study participants were not required to have hospital privileging in POCUS. The authors chose an initial sample size of 18 participants (6 from each site) based upon the relative homogenous sample, focused research questions [24], and sample size recommendations for qualitative studies [25].

**Table 1:** Characteristics of sites included in POCUS interview study

	Site 1	Site 2	Site 3
Practice Setting	Urban academic	Urban academic	Urban academic
Affiliated VA health center	Yes	Yes	Yes
Residency size^a^	85	104	46
POCUS Program			
Medical school curriculum	Yes	Yes	Yes
Residency Curriculum	Yes	Yes	Yes
Ultrasound Fellowship	No	Yes	Yes
Faculty Development Program	Yes	Yes	Yes
FTE for POCUS Program Director	Yes	Yes	Yes
Cart-based POCUS machines^b^	2	4	5
Handheld POCUS machines^b^	4	0	4

*Abbreviations*: *POCUS* point-of-care ultrasound, *VA* Veteran Affairs, *FTE* full-time equivalent

^a^Includes categorical, preliminary, and primary care residents

^b^Department-owned machines available for use by internal medicine faculty and residents

### Interviews

We conducted semi-structured, in-person interviews from July–August 2019. Interviews lasted 20–30 min and were recorded for transcription. Using a targeted needs assessment framework, interview questions explored content about the participants’ experiences and learning environment related POCUS [26]. The specific issues under investigation were motivators, facilitators, barriers, and professional engagement. Each interview topic started with a general, open-ended question, followed by probing questions (Additional file 1).

Questions exploring motivations were informed by the self-determination theory (SDT). SDT identifies 3 psychological factors that facilitate psychological growth and intrinsic motivation – autonomy, competency, and relatedness to the social environment [27, 28]. The interview guide included probing questions addressing each of these core motivational factors. The second interview question asked participants to describe barriers and facilitators to learning and integrating POCUS into their clinical routine. Probing questions were informed by practice implementation research and included questions exploring barriers related to knowledge, attitudes, and external factors [29]. The third question asked interviewees to reflect on how learning POCUS impacted their feelings of engagement or burn-out. Burn-out and engagement are overlapping concepts and are used as indictors of work-related well-being [30].

The interview guide was developed using an interactive process, including experts in POCUS and qualitative methodology. It was tested for clarity by conducting a pilot interview with an eligible faculty member. A recording of the pilot interview was reviewed by members of the research team, which did not result in any changes in the interview guide. Data from the pilot were not included in the final analysis.

### Analysis

Audio recordings were transcribed verbatim by a third-party service associated with the lead author’s institution using Descript online software (Descript, San Francisco, CA), resulting in 138 pages of single-spaced transcripts. A transcriptionist reviewed the transcripts for accuracy and removed identifying information. Two experts in qualitative methodology (RS and EB) analyzed transcript data using MaxQDA 2020 (VERBI Software, 2019, Berlin, Germany). We had no a priori hypothesis. The analysists used an inductive process to code the lines of text and identify emerging themes. First, each analyst independently coded transcripts from 2 interviews and compared results to create a common codebook. Then each expert coded half of the remaining interviews to identify themes that emerged from groups of codes. No additional themes were generated after analyzing the initial 18 interviews, therefore further interviews were not needed.

Study participants validated thematic results via member checking [31]. A summary report was sent to all participants via email. They were asked to review the report and evaluate for accuracy and completeness. Fourteen of 18 (78%) participants responded to member checking. Thirteen participants agreed with the summary report as written. One participant suggested clarifying language for one of the themes.

### Ethical considerations

Interviewees provided informed consent to participate in the study. Privacy was maintained by conducting interviews in private settings. Interview transcripts were de-identified to protect the anonymity of participants and no identifying data was used when reporting the results. Data was stored on an encrypted, password protected cloud drive managed by the lead author’s institution.

Reflexivity was managed by recognizing the power dynamics between researchers and participants [32]. Interviewers (CS, KB, KP) were leaders in their respective POCUS programs, which had the potential to influence participants’ responses. This was addressed by recruiting participants without hierarchical or managerial relationships with the researchers (i.e. colleagues of similar status). Part of the consent process included discussion that their involvement would not impact their standing within the POCUS program. The project was approved as exempt research by each site’s local Institutional Review Board (University of Nebraska Medical Center 278-19-EX, Oregon Health & Science University STUDY00016922, University of South Carolina Pro00089230).

## Results

Characteristics of the 18 participants are listed in Table 2. Qualitative analysis yielded 6 themes from the interview data: improved teaching performance, enhanced patient care, learning and curriculum needs, workflow and access, administrative support, and work engagement.

**Table 2:** Characteristics of 18 Physicians Participating in Interviews

Female Gender, n (%)	6 (33.3)
Median time since completing residency (IQR), yrs	5 (3.3–10)
Median time since first POCUS training (IQR), yrs	3 (1.3–5.8)
Clinical practice environment, n (%)	
Inpatient	12 (66%)
Hybrid/traditional	6 (33%)
Frequency of POCUS use, n (%)	
Less than weekly	6 (33.3)
Weekly	3 (16.7)
Several times per week	6 (33.3)
Daily	3 (16.7)
Comfort level using POCUS clinically, n (%)	
Uncomfortable	2 (11.1)
Neutral	8 (44.4)
Comfortable	8 (44.4)
Median clinical time with learners (IQR), weeks/year	12 (8–25)
Educational leadership role, n (%)	12 (66.7)
POCUS fellow, n (%)	2 (11)

*Abbreviations*: *POCUS* point-of-care ultrasound, *IQR* interquartile range

### Teaching performance

Interviewees felt POCUS augmented their teaching skills and enhanced their relationships with trainees. Integrating POCUS into their teaching made them feel valued and relevant as educators.

“If I look at my role as an educator, I feel like I'm able to offer something new that is needed in our specialty and for our learners.”

Participants also noted how POCUS can engage learners by using technology to integrate anatomy and physiology with bedside teaching:

“You can talk on rounds or you can do a sit down session or whatever but if you actually are at the bedside and you pull up the pictures and images especially when technology is involved like ultrasound is and they can draw on some of those anatomical things that they remember learning, I think that really draws them in.”

### Patient care

A second theme was the power of POCUS to improve patient care through patient engagement and enhanced clinical decision making. POCUS fostered a return to the bedside, resulting in more personal contact with patients. Participants also noted that POCUS images could be used as visual aides to help reinforce patient education.

“…it gives you a lot of time at the bedside with the patient which can be unusual the way we practice medicine. And then they like looking at their anatomy and learning about their own body.”

“I can show them, ‘Look, this is your heart. It's supposed to squeeze this much and it's only squeezing this much’ or, ‘Hey, look, there's fluid in here. There shouldn't be fluid in here.’ So even something as simple as that just having their visual feedback to the patient that can show them what we're doing and why.”

Interviewees reported that POCUS enhanced their clinical decision making by supplementing the physical exam and providing immediate, actionable data:

“I think one of the things that kept me going with it was the immediate reinforcement…if I saw something I can make a clinical decision and I could see whether that clinical decision was right or not…And I think I just really felt like in the past I wasn't able to make those ‘at the bedside’ decisions and now I was able to, so that was one of the very positive things.”

### Learning & curriculum design

Interviewees provided insight into what factors enriched their learning. First, a sense of community among POCUS learners helped maintain a fun, positive learning environment. This was reinforced by opportunities for peer-learning among faculty.

“I feel like we're all kind of learning at the same time, so that's nice…gathering our ultrasound images for review where several of us would get together and ultrasound patients together. That was actually fun. I enjoyed not feeling so isolated on my floor, away from the other hospitalists. So those were fun experiences.”

Second, interviewees noted the importance of expert coaches when learning POCUS. Having an experienced POCUS user provide ongoing quality assurance and constructive feedback on image acquisition and interpretation was considered important for developing confidence and independence.

“You have to obtain the images but you also have to review them with somebody who can say, ‘Well you were a little off axis, but you have enough here to have drawn the conclusion that you drew.’ You don't always have to have a perfect image and so that was a real big confidence builder for me both with regard to you know helping me with my technique but also helping me kind of understand what's good enough to answer the question.”

Conversely, lack of longitudinal support and feedback was a common barrier that limited participants’ confidence in supervising learners.

“I always could use more education and to build confidence in using it. Would I absolutely trust my own findings on ultrasound without verifying it with either someone who's better at ultrasound than me or verifying it with other studies? No, I wouldn't...not in my current level.”

Finally, interviewees suggested a graduated approach to learning POCUS. Interviewees suggested starting with straight-forward, normal exams before advancing to more challenging studies, as certain patient characteristics, such as obesity and immobility, were difficult for novice learners to navigate.

“Practicing on “normal” patients first just to work on again picture quality and acquisition and correct orientation of the probe and those kinds of things are the most helpful.”

“So those patients who are obese..I don't get a very clear picture where I can read the image with confidence then you know I'm not very good, so I have to improve my knowledge and skill in order to..read those not very optimal images.”

### Workflow & equipment accessibility

Minimizing disruption to established workflow was an important aspect of learning POCUS. Training flexibility was an important curricular design factor that allowed participants to more easily integrate learning into their unpredictable scheduling and minimize added workload. Novice users required more time to plan and perform POCUS exams, which could be difficult to manage with other competing responsibilities. Furthermore, practice inertia was difficult to overcome, as interviewees were not immediately in the habit of integrating POCUS into their daily routine.

“It's definitely not a habit or routine to think about ultrasound, so it's not going to be the first thing that comes to mind if I'm taking care of the patient that, so that is something if it became a habit or routine, then I definitely would use it more and then the more that I used it, the more confident I would become.”

An important element of workflow efficiency was machine accessibility. A primary barrier was limited availability and portability of cart-sized machines. Transporting and configuring these machines within patient rooms was sometimes cumbersome. Furthermore, machines were often communal devices stored away from patient care areas. Interviewees reported feeling frustrated when they went out of their way to get the machine, only to find it was unavailable.

“I can't find a machine. That's been like less than five times I'm sure. But that's tough because if you have the time and you have the energy to go get the machine and then it's not there…I think that having machines not available in all towers is tough.”

Conversely, hand-held devices were felt to facilitate learning through easier portability and accessibility.

“I can answer most of the questions with the handheld that I have and that also makes it a lot more easy and portable and it makes my chances of actually doing it a lot higher because I'm likely to have it on me. You know a lot of times if you're talking to the patient and you have a question you may or may not know if you want to do POCUS before you entered the room and so if you just have it around your shoulder, that makes it really easy just to turn it on versus going back and getting the machine and hauling it back is a barrier. So, I think for my purposes…the handhelds are great.”

### Administrative support

Another theme generated from interviews was the need for administrative support to finance and prioritize faculty development. This includes the need for dedicated time to practice nascent POCUS skills and avoid skills decay.

“I think that even as an institution that's strong in ultrasound we don't actually have a well-developed faculty development system to help faculty. We have plenty of training but just to use an example like the first number of years here I would get invited to training but they wanted me to pay for them. There also is no system that encourages ultrasound training at the cost of missing other responsibilities like no one at a high level saying we need everyone up to speed. Find a way to get people out of clinic because they all need to do ultrasound training at the faculty level.“

### Professional engagement

Participants felt learning POCUS enhanced their sense of engagement with their work and helped combated burnout. This was facilitated by several aspects of POCUS, including more direct-contact with patients, positive learner feedback, and the challenge of mastering a new skill.

“This is one procedure that at least combines the fact that I can do something with my hands, but also my head. It might be the only procedure I can think of that's not just a mindless route procedure that actually involves a lot of thought process while you're doing it. A lot of thinking. So, I do find it to be something that reminds me why I went into internal medicine and allows me a chance to learn something new. And every time I can learn something new, I become re-engaged with something that at times can get old.”

“I think that point of care ultrasound can...help fight against burnout I guess just because I feel like it's cool and it's innovative and that you know your learners like it, and your residents like it, your patients like it and so it can be pretty rewarding.”

## Discussion

In this study, IM clinician-educators describe the motivations, barriers, and facilitators to learning POCUS and integrating this new skillset into their clinical and teaching practices. Participants felt POCUS enhanced their teaching performance, patient care, and work engagement, while also providing important insights into how faculty development curricula can be designed to address the needs of practicing physicians (Table 3).

**Table 3:** Summary of themes and recommendations for POCUS faculty curriculum design

Theme	Recommendations
Improved teaching performance	Targeted recruitment of clinician-educators Integrate POCUS skills with traditional bedside teaching exams Leverage support from undergraduate and graduate medical educational leadership
Enhanced patient care	Highlight “return to the bedside” and potential to improve patient experience in faculty recruitment and advocating for resources with administrative leadership Prioritize curriculum content to address common real-world applications
Curricular needs	Community building among learners via peer-learning and communal experiences e.g. partner scanning, group image review, journal club, etc. Longitudinal training & feedback: Regularly scheduled sessions with experts (in-person or virtual). Quality assurance process with synchronous and/or asynchronous image review and feedback. Graduated skills training, progressing from simple to more complicated skills and exams
Workflow integration	Maximize portability and accessibility of equipment. Make handheld devices available and place shared devices in strategic locations, such as workrooms or patient care areas Integration of image archiving and documentation into existing systems and workflow Curriculum flexibility to support individualized learning and scheduling e.g. online lectures, access to simulation trainers, standing “office hours” for expert coaching, flexible deadlines for portfolio generation
Administrative support	Leverage support by considering how POCUS can address institutional needs and priorities for the hospital, department, and/or educational program Highlight potential of POCUS training to mitigate feelings of burnout Establish billing system for POCUS exams to offset resource needs Highlight potential for improved patient care outcomes e.g. diagnostic accuracy and procedural safety
Engagement with work	Promote potential to improve work engagement and combat burnout when recruiting faculty and soliciting administrative support.

This study adds to prior research in several ways. While previous studies have explored perceived barriers to POCUS, most have targeted residency program directors [13, 14, 16], which may not reflect the experiences of practicing physicians. A recent survey-based study of hospital-based internists identified primary barriers to learning POCUS, including lack of training, handheld devices, supervision, time, and quality assurance. The current study corroborates and expands on these findings by using qualitative methodology to explore “how” and “why” faculty physicians learn POCUS, including their motivations and professional engagement. While this study focused on IM faculty, we feel the findings are applicable to other specialties, such as Pediatrics [13] and Family Medicine [14], who face a similar challenge of expanding faculty expertise to develop, implement, and sustain residency POCUS programs.

Several of the themes generated from this study related to the need for longitudinal curricular support. In particular, the need for regular supervision, quality assurance, and feedback from experts is vital [33, 34]. Numerous studies have demonstrated that POCUS skills decay rapidly with disuse [35–37], which can be mitigated with longitudinal support [38–40]. Synchronous and asynchronous image review and feedback is important for skills advancement, as well as portfolio development to help meet privileging requirements. If local expertise is limited, learners may consider enrollment in training certificate programs through professional organizations [41–43]. Longitudinal curricula should also integrate instructional scaffolding, in which early learners receive the most support, which is gradually decreased as they attain competency. This concept also should influence the choice of scanning models. Study participants noted certain patient characteristics, such as obesity, were a common barrier to their learning. Curriculum designers may consider starting novices with simulation trainers and technically straightforward studies on healthy patients before advancing to more technically challenging patients. Lastly, the concept of peer-learning and sense of community around POCUS came up several times. POCUS directors can take advantage of this by intentionally building peer-support into longitudinal POCUS training. This may include peer scanning sessions, group image interpretation sessions, mentoring dyads, and informal social gatherings.

Streamlined integration of POCUS into existing clinical workflow is crucial to clinician-educators. Perhaps the most immediate way to accomplish this is through the use of handheld devices. These units are increasingly affordable, making it possible to provide dedicated devices for individual physicians or teams. That said, many programs will still rely on communal cart-based ultrasound machines. Inpatient physicians are often responsible for patients in multiple units across geographic locations, making machine accessibility a challenge. Recognizing this difficulty, our findings highlight the importance of keeping communal machines as conveniently located as possible, such as in workrooms or within patient care areas. Another strategy for encouraging POCUS practice is integration of documentation and image archiving workflow within existing clinical workflow to facilitate quality assurance while minimizing disruptions.

The need for administrative support is also vital to facilitating POCUS curricula for practicing internist. This includes financial support for training and equipment, but may also include providing protected time away from other clinical duties for novice POCUS users to practice their nascent skills. Other findings from this study may help justify this investment of resources. This includes the potential for improved clinical care, learner experiences, and patient education.

Lastly, the potential for POCUS training to enhance physician engagement and combat burnout is a powerful lesson. Participants noted that learning POCUS was fun, made them feel valued, and reinforced their motivations for practicing medicine (a return to the bedside). That said, care must be taken to ensure learning POCUS is not another task forced upon an involuntary audience. Recruitment of interested, self-motivated individuals, in combination with administrative support, may help sustain participants within POCUS faculty development programs.

This study had several limitations. The physicians interviewed were from academic centers with established POCUS programs, which may limit generalizability. Other limitations include the possibility that interview data was influenced by recall and social desirability biases.

We have started to make important changes to our POCUS curricula based upon the study results. For example, we are moving away from one-time, in-person didactics to online content to accommodate the demanding schedules of practicing physicians. We have also expanded the availability of handheld ultrasound devices and integrated image archiving systems to allow for longitudinal, asynchronous image review and feedback. Future research should explore the experiences of faculty in other disciplines and settings.

## Conclusion

This study provided a qualitative exploration of IM clinician-educators’ experiences learning POCUS, resulting in practical recommendations to help curriculum developers meet the unique needs of faculty learners. As POCUS becomes more ubiquitous in medical training, these data help contextualize the enthusiasm teaching physicians have for learning an innovative skill, while also highlighting challenges that should be proactively considered and mitigated.

## Supplementary Information

**Additional file 1.**

## Data Availability

The study data are available from the corresponding author by request.

## Abbreviations

IM: Internal Medicine
IQR: Interquartile range
POCUS: Point-of-care ultrasound
