# Supplementary material for: Motivations, barriers, and professional engagement: a multisite qualitative study of internal medicine faculty’s experiences learning and teaching point-of-care ultrasound
Source: BMC Med Educ. 2022 Mar 12;22:171. doi: 10.1186/s12909-022-03225-w (PMC8918294; doi:10.1186/s12909-022-03225-w)
Supplement: Supplementary file 1 — Additional file 1. [file 12909_2022_3225_MOESM1_ESM.docx]

Internal Medicine Faculty Experiences in Learning Point-of-care-ultrasound

Interview Guide

Interviewer: The purpose of this interview is to explore your experiences with the use of point of care ultrasound, sometimes referred to as “POCUS,” in order to inform future curriculum development. This interview is being audio recorded. We are asking for permission to use any part of this interview for research purposes, including scholarly presentations and publication. Your interview will be transcribed and de-identified to ensure that your statements remain anonymous. Your decision to participate is voluntary and will not affect your standing within the ultrasound program. Do you have any questions prior to starting?

Do you give consent to participate in this study? (After verbal agreement, proceed with questions)

1. What are your motivations for learning POCUS? Consider both internal and external motivating factors.
   1. Probe: Can you describe any experiences that positively and negatively impacted your personal interest, enthusiasm, or sense of independence in learning POCUS?
   2. Probe: Describe learning experience that most supported your development of POCUS skills?
   3. Probe: Describe how learning POCUS impacted your relationships with colleagues, students, and patients?
2. In thinking about your experiences in learning POCUS, are there any examples in which you wanted to perform a POCUS exam, but couldn’t because of some kind of obstacle or limitation?
   1. Probe: Have you experienced any barriers related to knowledge deficits?
   2. Probe: Have you experienced any barriers related to attitudes?
   3. Probe: Have you experienced any external barriers, such as factors related to resources, the environment, or patients?
      1. Interviewer Note: If lack of time described, explore specific contributors to time barriers
   4. Probe: What has or could help overcome these barriers?
3. Using your own definitions, how would you describe the impact that learning POCUS had on your feelings of burn-out or level of engagement?”
